# Functional diversity of Himalayan bat communities declines at high elevation without the loss of phylogenetic diversity

**DOI:** 10.1038/s41598-021-01939-3

**Published:** 2021-11-19

**Authors:** Rohit Chakravarty, Ram Mohan, Christian C. Voigt, Anand Krishnan, Viktoriia Radchuk

**Affiliations:** 1 Leibniz Institute for Zoo and Wildlife Research, Alfred-Kowalke Str. 17, 10315 Berlin, Germany; 2 Department of Animal Behaviour, Institute of Biology, Freie Universität Berlin, Berlin, Germany; 3 Department of Biology, Indian Institute of Science Education and Research (IISER) Pune, Pashan Road, Pune, 411008 India; 4 Present Address: Department of Biological Sciences, Indian Institute of Science Education and Research (IISER) Bhopal, Bhauri, 462066 India

**Keywords:** Ecology, Biodiversity, Biogeography, Community ecology

## Abstract

Species richness exhibits well-known patterns across elevational gradients in various taxa, but represents only one aspect of quantifying biodiversity patterns. Functional and phylogenetic diversity have received much less attention, particularly for vertebrate taxa. There is still a limited understanding of how functional, phylogenetic and taxonomic diversity change in concert across large gradients of elevation. Here, we focused on the Himalaya—representing the largest elevational gradients in the world—to investigate the patterns of taxonomic, functional and phylogenetic diversity in a bat assemblage. Combining field data on species occurrence, relative abundance, and functional traits with measures of phylogenetic diversity, we found that bat species richness and functional diversity declined at high elevation but phylogenetic diversity remained unchanged. At the lowest elevation, we observed low functional dispersion despite high species and functional richness, suggesting a niche packing mechanism. The decline in functional richness, dispersion, and divergence at the highest elevation is consistent with patterns observed due to environmental filtering. These patterns are driven by the absence of rhinolophid bats, four congeners with extreme trait values. Our data, some of the first on mammals from the Himalayan region, suggest that in bat assemblages with relatively high species diversity, phylogenetic diversity may not be a substitute to measure functional diversity.

## Introduction

Elevational gradients are characterized by stark changes in climate and vegetation over short spatial scales, which, in turn, dramatically shape biodiversity^1,2^. For example, species richness (SR) exhibits well-described patterns across elevations: SR either declines linearly with elevation, or exhibits a mid-elevation peak^3^. However, studies have increasingly recognized that focusing only on SR provides a coarse quantification of biodiversity^4–6^, because knowing how many species a location supports, does not provide ecological information about each species, or the traits enabling them to inhabit that location. Therefore, in addition to SR, macroecological studies are increasingly measuring two other aspects of biodiversity: functional diversity (FD) and phylogenetic diversity (PD). FD deals with traits that allow organisms to perform their range of functions in the ecosystem^7^, whereas PD assesses the diversity of evolutionary relationships among species in a community^8^. Such complementary consideration of different aspects of biodiversity enables a comprehensive understanding of community assembly, which can help to better predict the impact of climate change on these communities^9^.

Biodiversity is structured along natural environmental gradients by the interplay of abiotic and biotic factors, resulting in certain observable patterns in FD and PD^10^. Community assembly theory^11,12^ predicts that harsh environmental conditions (typically higher elevations in mountains) support fewer species because the abiotic environment may select for certain traits that allow species to cope with harsh environmental conditions^13^. This so-called abiotic or environmental filtering^11,14^ reduces the dispersion of functional traits within a community. On the contrary, competitive interactions often result in increased trait dispersion, usually in regions with benign environmental conditions and high diversity (typically low elevations in mountains), because each species specializes to its specific ecological niche^10,13^. If traits are phylogenetically conserved, PD varies congruently with FD^13^. Dispersal and colonization may also influence PD. For example, high rates of in situ diversification or immigration of multiple taxonomic lineages may increase PD^15^. Thus, observing the patterns of taxonomic, functional and phylogenetic diversity in concert allows us to infer the mechanisms behind community structuring^13,16^.

Most of our knowledge of community structure along elevational gradients comes from studies on birds and plants. Recent meta-analyses of global functional and phylogenetic data of birds, independently found no evidence of uniform patterns of FD and PD across elevations^17,18^. Tropical lowlands and temperate highlands are functionally overdispersed whereas tropical highlands and temperate lowlands exhibit functional clustering and redundancy. Interestingly, in tropical lowlands, closely-related bird species have similar traits, and this pattern is less evident as elevation increases^18^. Despite the immense value of these studies in understanding how community composition varies with elevation, two key limitations emerge. The first is that most findings are based on published occurrence data or museum specimens that lack information on species’ relative abundance or co-occurrence^10^. Such use of occurrence data may lead to biased biodiversity estimates, because they usually do not correct for detection probability, which may differ among species with different traits^19^. The second limitation is that studies of community structure have primarily focused on a few taxa, and not on those that are elusive and hard to sample, such as bats. Studying a wider range of taxa may shed light on the generality of observed elevational patterns in biodiversity.

Bats form the second most speciose mammalian order^20^ and are therefore an excellent system to investigate community structure along environmental gradients. Furthermore, bats are bioindicators of climate change^21,22^ and their capability of powered flight may enable them to shift their elevational ranges within short time periods^23,24^. Field studies along the Andean slopes indicate that species richness decreases with increasing elevation^25^. A global model based on regional and local climatic factors predicts that the SR of bats decreases linearly with elevation in mountains with warm and wet bases, whereas mid-elevation peaks are predicted in mountains with dry and arid bases^3^. However, the relationship of FD and PD to changes in SR remain poorly explored. Previous trait-based and phylogenetic studies of bats along elevational gradients have returned inconsistent results. FD has been observed to decrease^26^ or increase^4^ only at the highest elevations. Similarly, different studies found that environmental filtering leads to different traits dominating assemblages at high elevations: wing manoeuvrability^27^ vs smaller body sizes^4^. The variation in PD with elevation is very poorly understood in bats. The only study that investigated this aspect of diversity found an increase in phylogenetic dispersion above 2500 m^4^. These inconsistencies highlight the differences among mountains and regional species pools necessitating more studies across the world if we are to obtain a general coherent picture of how bat diversity changes with elevational gradients.

In this study, we investigated the variation of taxonomic, functional and phylogenetic diversity of bats across a 2000 m elevational gradient in the Himalaya. Although the Himalaya have the highest mountain peaks in the world, their elevational floral and faunal biodiversity patterns, and the potential mechanisms behind these patterns have received little attention. With the exception of a few studies on birds^17,18^, plants^28,29^ and insects^30,31^, most other taxa, including bats, remain poorly studied. The Himalaya are warming considerably faster than the global average^32^ leading to range shifts in species^33^, and thus a comprehensive understanding of biodiversity patterns is particularly relevant. Specifically, assuming phylogenetic conservatism of traits, we predict that (a) measures of functional and phylogenetic richness decrease with elevation; (b) the high elevational community is functionally and phylogenetically underdispersed due to extreme environmental conditions that select for certain traits, and (c) the lower elevation community exhibits greater functional and phylogenetic dispersion, as a result of more available niches, and, potentially, stronger competitive interactions. Alternatively in the absence of phylogenetic conservatism, we expect that high elevation communities are phylogenetically overdispersed if distantly-related species are characterised by similar physiological tolerance. By incorporating field data on species abundances from mistnetting and acoustic sampling methods, we additionally investigated functional evenness and divergence, in contrast to previous studies that lacked primary field data. To our knowledge, with the exception of birds, no study has hitherto been conducted on functional and phylogenetic diversity of any vertebrate taxon in the Himalaya. Our study thus provides valuable insight on community composition across a Himalayan elevational gradient.

## Methods

### Study area and sampling locations

**Figure 1:**
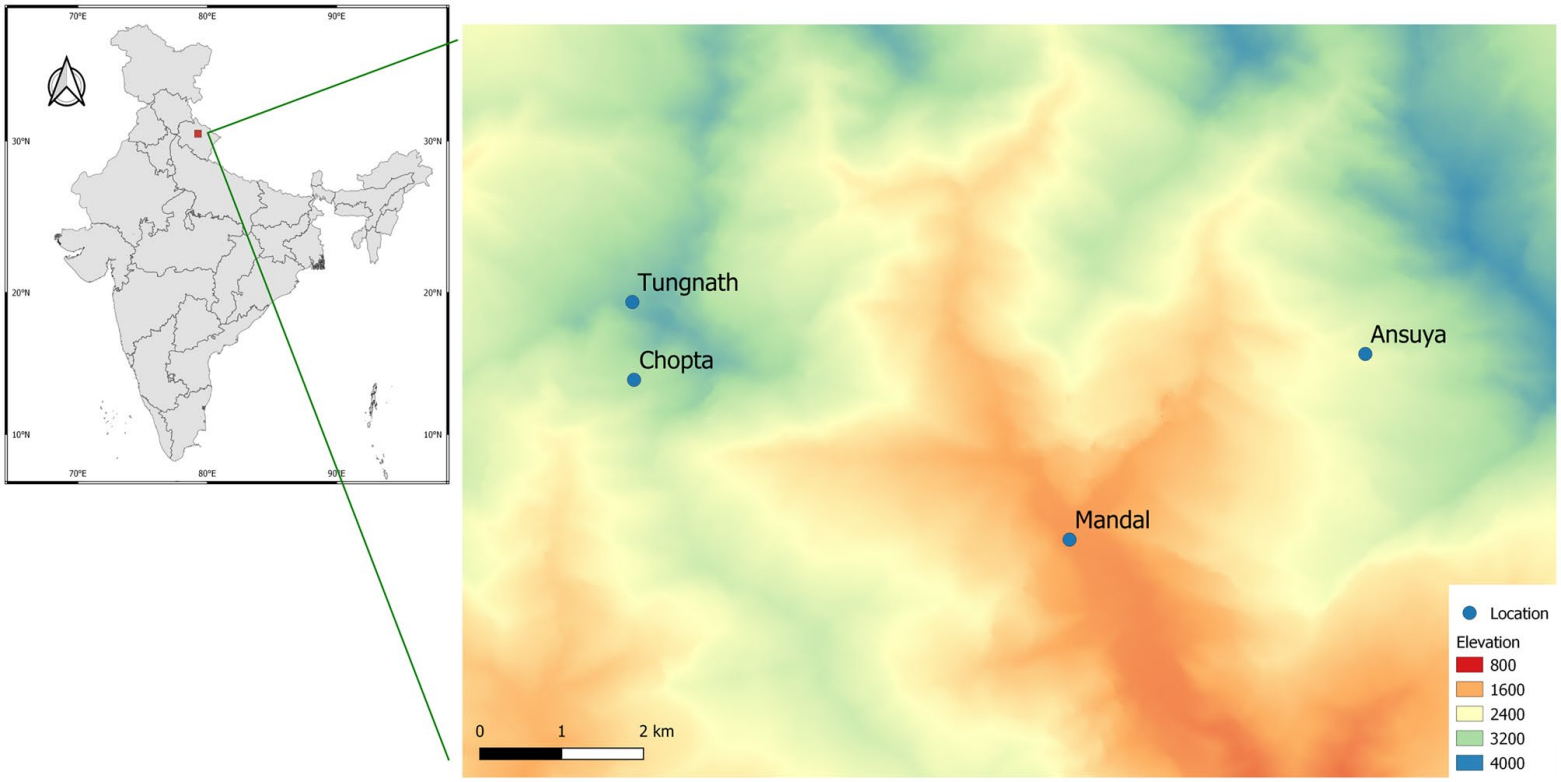
Map of India showing the location of the study area, Kedarnath Wildlife Sanctuary, and the sampling locations within the study area. Elevation is in m asl. The map was created using QGIS (v 3.6.3-Noosa) (QGIS Geographical Information System, www.qgis.org). Please note that the geographical boundaries represented in the map may contain areas considered disputed.

We conducted this study in Kedarnath Wildlife Sanctuary (30° 25′–30° 41′ N, 78° 55′–79° 22′ E), located in Uttarakhand state in the western Himalayas of India. This sanctuary covers a broad elevational gradient from 1400 to 4000 m above sea level (asl) (Fig. 1), with corresponding changes in habitat types: from Himalayan moist temperate forests dominated by *Quercus* spp. at low elevations, to sub-alpine forests dominated by *Rhododendron* spp. and alpine meadows at high elevations^34^. This sanctuary is known to harbour 26 species of bats^35^.

We sampled at four locations spanning an elevational gradient of 2200 m. Sampling points within each location were spread across the elevations mentioned in parentheses: Mandal (1500–1800 m), Ansuya (2000–2200 m), Chopta (2700–3000 m) and Tungnath (3300–3700 m) (Fig. 1). Sampling was conducted between late-March and mid-May in 2018 and 2019, starting at lower elevations and then moving to higher elevations. This sampling duration coincides with summer in the Himalaya. To comprehensively sample the bat diversity, we employed a combination of automated ultrasonic recorders and capture sampling using mist-netting. Fieldwork was approved by the Internal Committee for Ethics and Animal Welfare, Institute for Zoo and Wildlife Research (approval no. 2018-06-01), and conducted under a permit issued by the Uttarakhand State Forest Department, Government of India (permit no. 2261/5-6).

### Sampling strategy

For acoustic sampling, we placed full spectrum passive ultrasonic recorders (SongMeter SM4BAT, Wildlife Acoustics, Maynard, MA, USA) in different habitat types (open, forest edge, and forest) at each elevation (hereafter, “passive recordings”). The recorders were programmed to record bat calls for two consecutive nights at each sampling point, from dusk to dawn (9–10 h/night), using a sample rate of 500 kHz/s, an amplitude threshold of 16 dB and a frequency threshold of 5 kHz. The dominant habitats at Ansuya and Tungnath are montane evergreen forests and alpine meadows respectively, therefore only these habitats were sampled at these elevations. The exact number of sampling points per habitat for each elevation is given in Table S1. On separate days after completing acoustic sampling at a site, we set up nylon and monofilament mist nets of 4, 6 or 9 m length, 16 × 16 and 19 × 19 mesh sizes (Ecotone GOC, Sopot, Poland) for four hours following dusk (starting between 18.30 h in early summer and 19.30 h in late summer). The captured bats were handled and measured following the guidelines of the American Society of Mammalogists^36^. To further refine identification in light of the paucity of taxonomic knowledge in the region, we collected only one specimen each of taxonomically-challenging species in accordance with our field research permit. We measured body mass (accuracy 0.1 g) using a spring balance (Pesola, Schindellegi, Switzerland), and forearm length (accuracy 0.01 mm) with vernier calipers (Swiss Precision Instruments SPI Inc., Melville, NY, USA). Next, we gently stretched the left wing and placed the live animal perpendicular to the background of a graph sheet of 1 × 1 cm grids. We photographed the outstretched wing using a Nikon D3400 DSLR camera at 55 mm zoom from a distance of about 90 cm. Subsequently, we released the bats and recorded their echolocation calls at a distance of 5 to 10 m using a handheld ultrasonic detector (Anabat Walkabout, Titley Scientific, Brendale, QLD, Australia) and saved them as audio files of .wav format. These recordings (henceforth referred to as “reference recordings”) formed the dataset used to develop a call library for identification.

### Call classifier and analysis of passive recordings

Reference recordings from 2018 and 2019 were labelled using Raven Pro 1.5 (Cornell Lab of Ornithology, Ithaca, NY, USA) to generate a dataset of acoustic parameters for identification. We visualized calls using a spectrogram with Hanning window, size 1024 samples with 95% overlap. From each recording, we selected 10 clear pulses and measured the following parameters: average peak frequency, maximum peak frequency, centre frequency, minimum peak frequency, peak frequency at the start and end of the call, bandwidth at 90% peak amplitude, average entropy, and call duration. All frequency variables were measured in Hz and time variables in ms. We used the peak frequency contour to determine start and end frequencies and also used bandwidth at 90% peak amplitude because higher frequencies attenuate quickly with distance from the emitting bat (causing changes to the bandwidth), and these measures are therefore more reliable in field circumstances. Using this labelled call library as a training dataset, we trained a fine K-nearest neighbours classifier using supervised learning within the ‘Classification Learner’ app in MATLAB (Mathworks, Inc., Natick, MA, USA). We further employed fivefold cross-validation to obtain estimates of the accuracy of each classifier in assigning calls to species. Using these pairwise values of relative accuracy (%), we generated confusion matrices for these classifiers where the species identities were represented in the columns and rows as ‘True’ and ‘Predicted’ classes, respectively. Any species with classification accuracy below 85% was clubbed with possible confusion species into a “sonotype”, to improve accuracy of the classifier in the most conservative way possible (Fig. S4). The complete list of sonotypes and their mean echolocation call parameters is presented in Table 1. The classifier identified these sonotypes with > 80% accuracy, with the exception of *Miniopterus* and the *Plecotus* type B call (which, however, we could manually identify because of their call structures and frequencies). For all subsequent analyses on functional diversity and phylogenetic diversity, we used these sonotypes to ensure accurate identification.

**Table 1:** Trait matrix of the sonotypes in our assemblage (FA in mm; fmaxe, pfc.min, and pfc.max in kHz; Duration in ms).

Sonotype	FA	AR	WL	I	fmaxe	pfc.min	pfc.max	Duration
*Arielulus circumdatus-Mirostrellus joffrei-Nyctalus leisleri* (AMN)	39.06	7.77	11.86	0.93	35.56	31.31	67.65	7.13
*Barbastella darjelingensis* (Bdar)	39.49	5.72	8.55	0.7	34.83	24.74	41.34	5
*Eptesicus-Hypsugo* (EH)	49.52	7.14	9.48	1.17	29.19	22.81	47.57	8.02
*Miniopterus fuliginosus* (Mful)	49.15	6.89	9.4	0.82	49.31	48.09	86.91	7
*Myotis muricola* (Mmur)	36.38	6.79	5.43	1.19	53.06	46.32	96.19	3.92
*Myotis sicarius-Pipistrellus* cf. *ceylonicus* (MP)	34.71	7.03	6.84	1.1	40.03	36.98	70.39	5.69
*Myotis longipes-Submyotodon caliginosus* (MS)	34.85	6.95	5.84	1.34	62.93	57.6	102.08	4.27
*Murina aurata-M. huttoni* (Murina)	30.29	6.22	6.51	1.5	83.2	70.8	122.24	2.62
*Plecotus homochrous-P. wardi* (Plecotus)	40.03	6.6	6.1	1.68	36.72	28.95	43.85	2.7
*Rhinolophus lepidus* (Rlep)	37.47	6.78	6.51	2.52	97.93	81.93	98.7	27.3
*Rhinolophus luctus* (Rluc)	71.06	6.29	11.26	2.35	31.25	27.78	31.25	49.46
*Rhinolophus pearsonii* (Rpea)	53.77	6.12	8.27	2.2	59.51	50.5	59.81	31.68
*Rhinolophus sinicus* (Rsin)	48.81	6.14	10.87	1.79	84.06	73.7	84.34	32.4

Species means of all traits were used to run a PCA and the first four PCs were used to calculated FD indices.

Next, we analysed the passive recordings manually in Raven Pro. We labelled calls in subsets of 15 min per hour of the passive recordings. For each hour, the 15-min subsets were in the time windows 0–5 min, 20–25 min and 40–45 min, so as to spread out our sampling window across the hour. Following labelling, we obtained sonotype IDs using the classifier, and then verified them manually by visual comparison to the call library to improve discrimination. For every 5-min interval, we made a presence-absence matrix where 1 indicated the presence of a sonotype and 0 indicated its absence. The number of 5-min intervals in which a sonotype was detected (hereby “acoustic detections”) was summed up for each sampling point. We measured the relative abundance of sonotypes as the proportion of its total number of acoustic detections relative to the total number of acoustic detections of all sonotypes in a given elevational location. The use of such a presence-absence framework is akin to ‘Acoustic Activity Index’^37^ which represents a relatively less biased index of activity that is less affected by differences in vocal behaviour and echolocation frequencies of different species of bats.

### Assessing detectability

To assess the completeness of our species inventory, we estimated the species richness of each sampling point using the first-order Jackknife Estimator (Jack 1)^38^. Jack 1 is a nonparametric procedure for estimating species richness using presence or absence of a species in a given plot rather than its abundance^39^. Mean species detectability was calculated as the ratio of the observed to estimated species richness for different sampling point-year combinations^40,41^. We then assessed whether this mean species detectability depended on the habitat type, year, and location by fitting a linear model with the above-mentioned variables as fixed factor predictors and the mean detectability as a response. We also determined species-level detectability by following the approach of Kéry and Plattner^42^. If a sonotype was detected by mistnetting or acoustic sampling in sampling event *i*, we modelled its probability to be detected in sampling event *i* + 1. For each sonotype, we fitted a generalized linear mixed-effects model (logit link and binomial error distribution) with detection/non-detection as the response variable, and habitat type, location, and year as the fixed factor predictors. Site and species were included as random intercepts. The significance of the fixed effects was assessed with the Likelihood Ratio Test. This test allows one to choose the best of two nested models by assessing the ratio of their likelihoods. The significance of the random effect (species) was assessed by applying a parametric bootstrap (number simulations = 100) to the model with and without the random effect, using the function bootMer of ‘lme4’ package. In short, a parametric bootstrap consists of fitting the model to the data and bootstrapping the obtained residuals. For these and other statistical analyses we used R version 4.0.2 (R Core Team 2020).

### Taxonomic diversity

We calculated rarefied incidence-based species richness (SR) and Simpson diversity extrapolated to 50 sampling events (the number of sampling events in Mandal) using the ‘iNEXT’ R package^43^. The calculations were performed on a sonotype-by-sampling point presence-absence matrix with detections from both acoustic sampling and mistnetting pooled together. In the matrix, columns represented sampling units (Night 1, Night 2 and so on) and rows represented sonotype. By using sonotypes instead of species, we likely underestimated the SR, but this underestimation was uniform across elevations and is unlikely to change the pattern of SR with elevation.

### Functional diversity

Our functional trait matrix (Table 1) comprised seven morphological and acoustic traits involved in guild classification, foraging and micro-habitat preferences (abbreviation followed by units): forearm length (FA, mm), aspect ratio (AR), wing loading (WL, N/m^2^), tip-shape index (I), echolocation peak frequency/frequency of maximum energy (FmaxE, kHz), minimum and maximum frequencies of the peak frequency contour (pfc.min and pfc.max, kHz) and call duration (D, ms). FA was measured in the field using vernier calipers. We used ImageJ (National Institutes of Health, Bethesda, MD, USA)^44^ to measure total wing area, areas of hand and arm wings and the wingspan from the standardised wing photos that were taken in the field. We calculated AR, WL, and I from these measurements following the equations given in Norberg and Rayner^45^. AR and WL both represent parameters that are correlated with flight aerodynamics and behaviour. I is influenced by the shape of the wing tip where values of 1 and above indicate broad, triangular tips, while those below 1 indicate acute wing tips. The four acoustic traits represent the shape of the echolocation call and they were measured from the reference recordings using Raven Pro, as described above.

We first calculated the means for each of the seven traits across all species within a sonotype (thus obtaining one average trait value for each sonotype) (Table 1) and then used those to compute four multivariate functional diversity (FD) indices: functional richness (FRic), divergence (FDiv), evenness (FEve)^46^, and dispersion (FDis)^47^, using the function dbFD() in the ‘FD’ R package^47^. Our FD measures are unlikely to be underestimated due to the pooling of species into sonotypes because these species were similar in acoustic and morphological traits. FRic is the convex hull volume of the traits of species present in a community, measured in the multidimensional trait space. This measurement is not weighted by abundance, relative abundance or biomass of the species in the community, but, it is standardised such that it ranges from 0 to 1. FDiv reflects the distribution of abundance across taxa (sonotypes in our case) in the functional space. High FDiv means the taxa with extreme trait values are more abundant in a community whereas low FDiv means that those with the trait values close to the centre of the functional space are more abundant^48^. FEve, on the other hand, measures the evenness in the abundance distribution of taxa in the functional space. FEve is high when all taxa have similar abundances, and it is low when some functional groups are abundant while others are rare^48^. Lastly, FDis is measured as the mean distance of all taxa to the abundance-weighted trait community centroid. We performed two sets of analyses: one using the number of mistnet captures as a proxy for relative abundance, and another using the number of detections of different sonotypes in 5-min intervals in the passive recordings as a proxy of relative abundance. We did not pool acoustic detections and mistnet captures as they have inherently different detection probabilities and measure different entities (relative number of detections vs. number of captured individuals). Owing to rhinolophid bats at lower elevations being taxonomically and functionally different from the remaining species pool, we performed another set of FD calculations, excluding the four rhinolophid species and using acoustic detections as relative abundance. One species, *Tadarida teniotis* was commonly detected at all elevations on acoustic recorders, but we were unable to capture it as it foraged high above the ground, and thus were unable to collect morphological trait data. Additionally, in using acoustic detections as a measure of relative abundance, we had to exclude the non-echolocating pteropodid bat *Sphaerias blanfordi* which was caught only once at Chopta. Therefore, our FD values are likely systematically underestimated across all elevational communities, which does not affect the comparison of community composition across elevations.

### Phylogenetic diversity

Using the nexus file of a published phylogeny^49^, we pruned the tree to represent species in the 14 sonotypes. For each of these types, we chose the species most commonly mist-netted as representative of its group. Published DNA sequences are lacking for some of the species in this region, so we chose their closest relatives from the phylogeny instead. Thus, we made the following replacements: (a) *Nyctalus leisleri* represented the AMN sonotype, (b) *Eptesicus serotinus* represented the EH sonotype, (c) *Murina aurata* for Murina sonotype, (d) *Myotis longipes* for MS sonotype, (e) *Pipistrellus javanicus* for MP sonotype, and (f) *Plecotus turkmenicus* for Plecotus sonotype. After pruning the tree, we calculated three indices of phylogenetic diversity using the ‘picante’ R package^50^: Faith’s phylogenetic diversity (PD), Mean pairwise distance (MPD) and Mean nearest-taxon distance (MNTD). Faith’s PD is a measure of phylogenetic richness which is obtained by summing the branch lengths of the tree connecting the species in the community. MPD and MNTD measure phylogenetic dispersion of communities; whereas MPD measures the average phylogenetic distance among all the taxa in a community, MNTD measures the same for the nearest neighbouring taxa^51^. We weighted MPD and MNTD by relative abundance of the sonotypes in each community (like FD, the number of detections in five-minute intervals in the passive recordings was used as a proxy of relative abundance).

### Null model testing

As FD and PD are strongly correlated to species richness^52^, we used a null model to assess whether the observed was significantly different than expected due to chance alone. We produced the null distribution of each FD and PD index by randomizing the community matrix 999 times using the ‘independent swap’ method^53,54^, so as to preserve the species richness at each site and the number of sites in which each species can be found. Our randomization was further constrained by elevation, so that the abundances were randomized among the sampling points within each elevation. The null model allows for calculation of an effect size (difference between the observed value and mean of the null distribution). Given the range of FD and PD values, the effect sizes are not comparable across communities with vastly different species richness^55^. Therefore, standardized effect sizes (SES) of each index were calculated at each site as the difference between the observed value and the mean of the null distribution, divided by the standard deviation of the null distribution. SES > 1 and SES <  − 1 indicate that a given index is significantly higher and lower (respectively) than the null model. Tungnath was excluded from the FD analyses due to inadequate number of sampling points (only two) for randomizations. We used a generalised linear model (GLM) to assess the change in each index with elevation. Tukey’s Honest Significant Difference (HSD) test was subsequently used to compare the means.

### Ethics declaration

Fieldwork was approved by the Internal Committee for Ethics and Animal Welfare, Institute for Zoo and Wildlife Research (approval no. 2018-06-01), and conducted under a permit issued by the Uttarakhand State Forest Department, Government of India (permit no. 2261/5-6).

## Results

**Figure 2:**
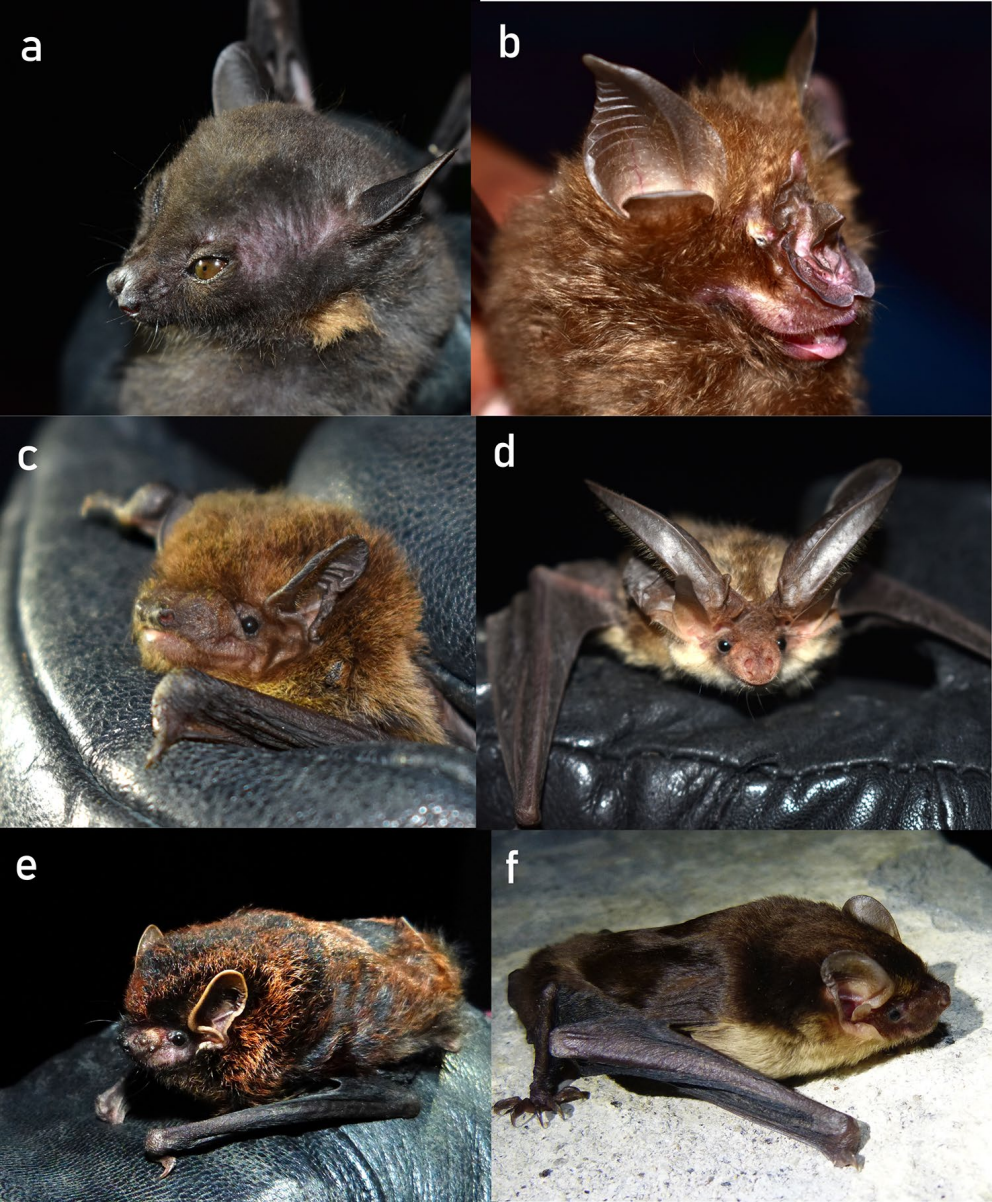
Portraits of some bat species found in Kedarnath Wildlife Sanctuary that represent different functional and phylogenetic groups. (**a**) *Sphaerias blanfordi*, (**b**) *Rhinolophus pearsonii*, (**c**) *Pipistrellus* cf. *ceylonicus*, (**d**) *Plecotus wardi*, (**e**) *Arielulus circumdatus* and (**f**) *Mirostrellus joffrei*. All photos by the first author.

We recorded a total of 23 bat species in 15 genera and five families (Pteropodidae, Rhinolophidae, Molossidae, Vespertilionidae and Miniopteridae) across the elevational gradient in Kedarnath Wildlife Sanctuary. Of these, *Tadarida teniotis* was only recorded through acoustic sampling whereas *Sphaerias blanfordi*, a non-echolocating fruit bat of the family Pteropodidae, was only caught in a mist net. Three species: *Hypsugo affinis*, *Myotis sicarius* and *Miniopterus fuliginosus* were not recorded in a recent survey^35^. Select bat species from the study area that belong to different functional groups are depicted in Fig. 2.

### Detectability

The median detection probability across species, combined across all sampling sites was 0.8 (Fig. S1). We found no significant effect of location (F = 0.44, df = 3, p = 0.73), year (F = 0.95, df = 1, p = 0.34) and habitat type (F = 1.47, df = 2, p = 0.25) on the mean species detection probability. We did not find a significant effect of species on species-level detection probability. However, habitat type affected species-level detection probability ($\chi$^2^ = 7.4, df = 2, p = 0.025), being lowest in the forest habitat and high in both open habitat and at forest edges (Fig. S2). In other words, any given species was less easily detected in forest habitats.

### Effect of elevation on taxonomic, functional and phylogenetic diversity

**Figure 3:**
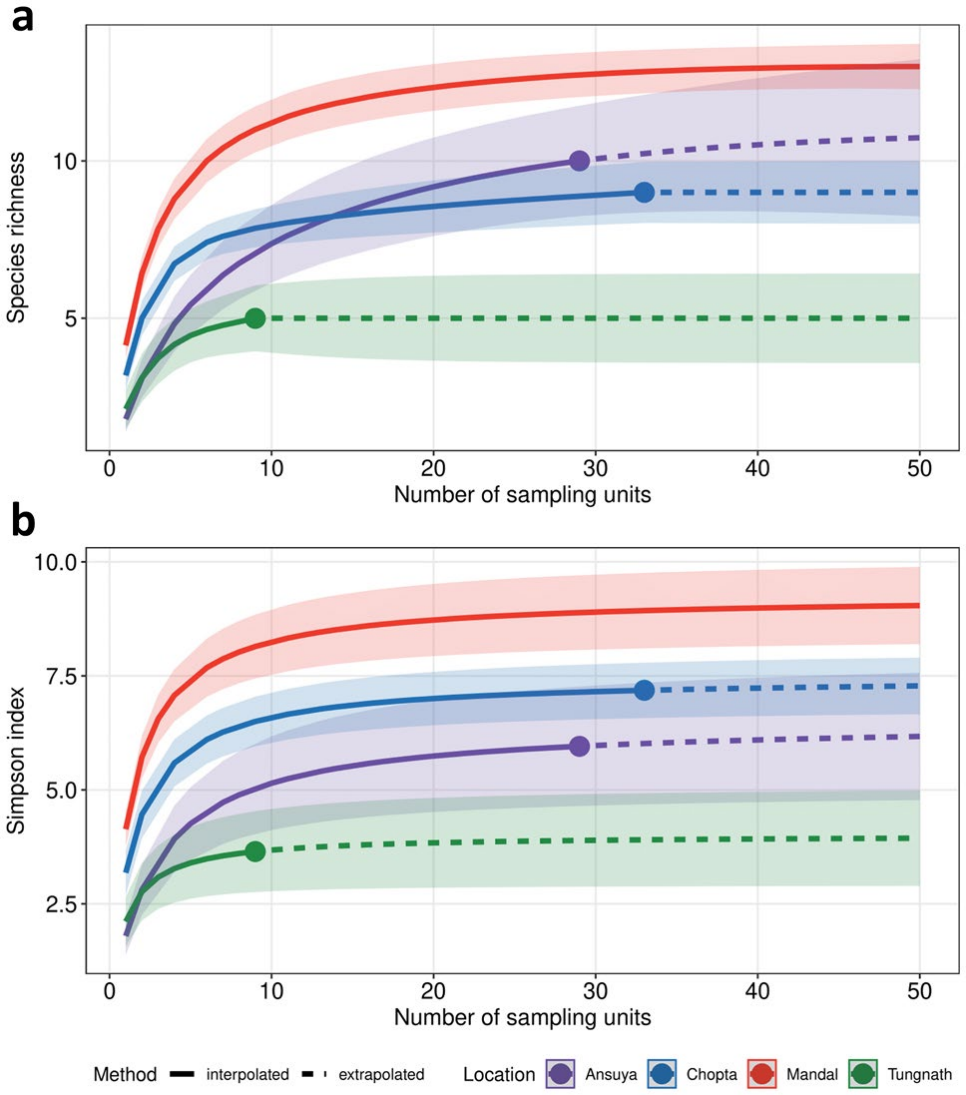
Rarefaction curves of (**a**) cumulative species richness and (**b**) Simpson diversity across all elevational communities plotted against the number of sampling nights. Each location is represented by a different colour. The symbols represent the number of sampling nights at each location. Species richness of all locations is extrapolated to the number of sampling nights at Mandal (shown by the dotted lines).

**Figure 4:**
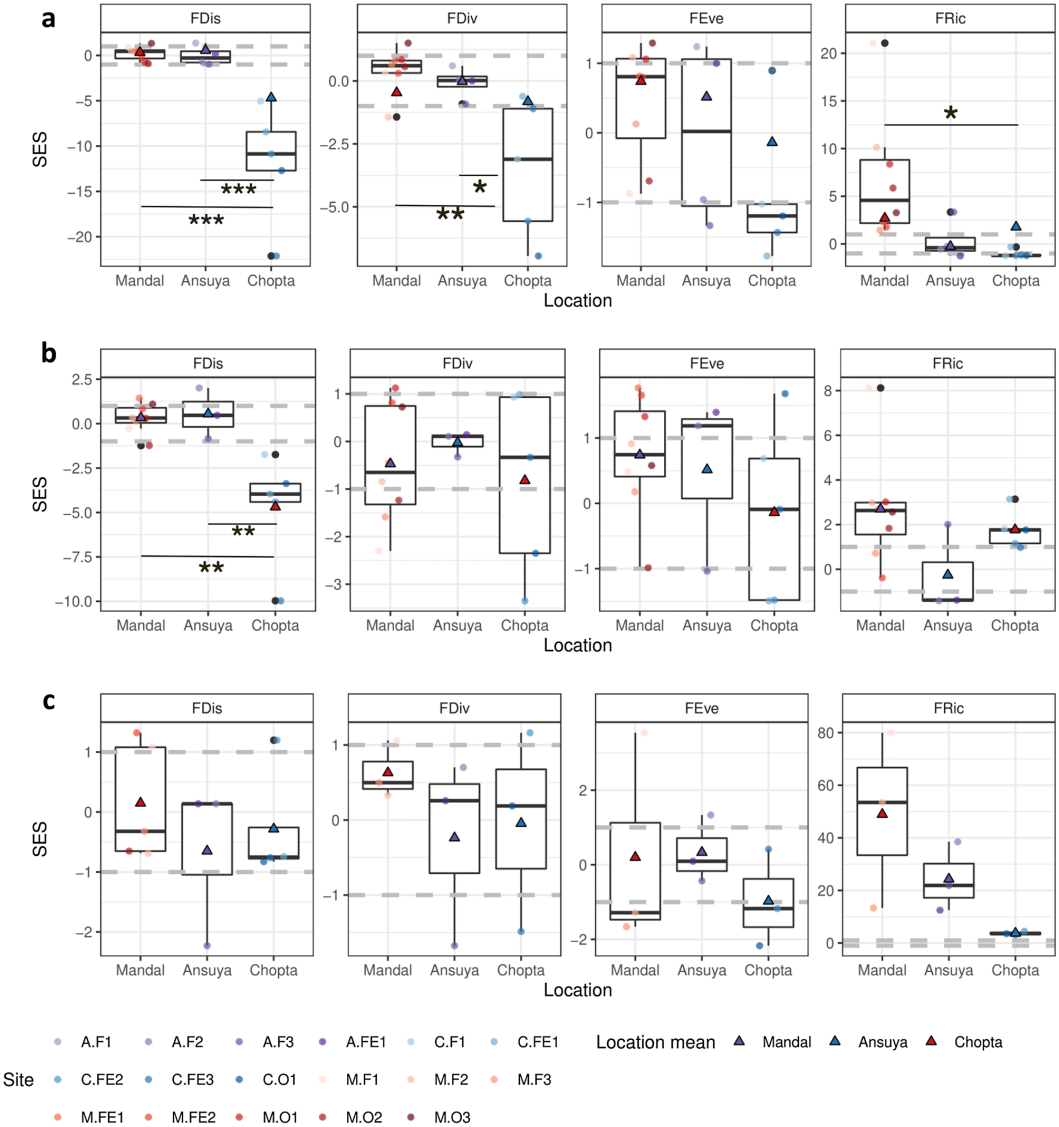
SES values of different elevational communities for corresponding FD indices obtained from constrained randomizations with 999 iterations; measured using (**a**) acoustic detections as relative abundance for all species/sonotypes, (**b**) acoustic detections as relative abundance excluding rhinolophid bats, and (**c**) mistnet captures as relative abundance for all species/sonotypes. |SES|= 1 and above are significantly different from the null model (for more details please see “Methods” section). Sampling points are denoted by dots and mean FD values by triangles within the boxplots. Lines connecting different elevations and *represent elevations with significantly different means as assessed from Tukey’s HSD test. *p < 0.05, **p < 0.01, ***p < 0.001.

Species (sonotype) richness (SR) decreased with increasing elevation. Simpson’s diversity was highest at Mandal, followed by Chopta, Ansuya and finally, Tungnath (Fig. 3). FD decreased significantly only at Chopta (Fig. 4), the highest elevation considered in the analysis. When calculating FD indices using acoustic detections as a measure of relative abundance, all FD indices at Chopta were significantly lower than expected under a random null community (SES < 1; Fig. 4a). FDis and FDiv at Chopta were also significantly lower than in Mandal and Ansuya (FDis Tukey’s HSD, p < 0.001; FDiv Tukey’s HSD, p < 0.05) (Fig. 4a). FRic at Mandal was significantly greater than expected under the null model (SES > 1). However, when we repeated the analysis by excluding the rhinolophids, the differences in FD indices across elevations were insignificant (Fig. 4b). Mean and median of FRic dipped at the mid-elevation and both Mandal and Chopta showed higher values than expected under a null model. Only FDis at Chopta was significantly lower than the null model and was also significantly lower than that in Mandal and Ansuya (Tukey’s HSD: p < 0.01). We found no effect of elevation on FD when measuring FD indices using mistnet captures as relative abundance (Fig. 4c).

**Figure 5:**
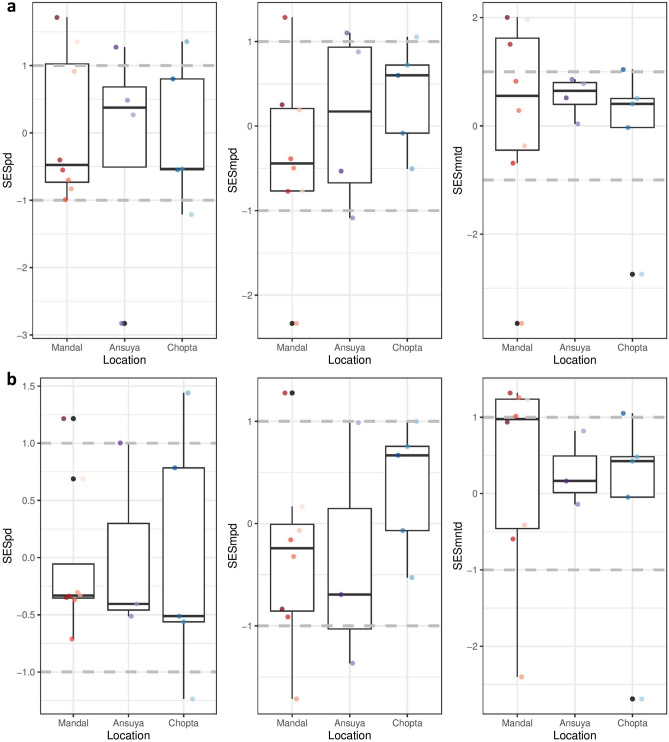
SES values of different elevational communities for corresponding PD indices obtained from constrained randomizations with 999 iterations; measured using acoustic detections as relative abundance for all species/sonotypes. (**a**) For all species, (**b**) for species excluding rhinolophids. |SES|= 1 and above are significantly different from the null model (for more details on SES see “Methods” section).

In contrast to FD indices, PD indices were not significantly affected by elevation irrespective of whether rhinolophid bats were considered in the analysis or not (Fig. 5). Faith’s PD, MPD and MNTD of all communities were not different from the random expectation (SES between − 1 and 1).

## Discussion

Our study combined field data on species occurrence, abundance, and functional traits with analyses of phylogenetic diversity in the first comprehensive study of elevational diversity patterns in Himalayan bats. We predicted that SR, FD and PD would decline with elevation, based on biogeographic studies in diverse taxa^3,17,18,26^. Assuming phylogenetic conservatism of traits, we expected FD (specifically FRic and FDis) and PD to be underdispersed (lower than the null expectation) at the highest elevational location, Chopta, and overdispersed (higher than the null) at the lowest elevational location, Mandal^10^. Contrary to our initial predictions, we uncover divergent trends in species (sonotype) richness (SR), functional diversity (FD), and phylogenetic diversity (PD) across this 2000 m gradient, with only FD being significantly lower than expected under random expectation at Chopta. Additionally, we found higher than expected FRic but not FDis at Mandal, the lowest elevation community. At the same time, PD did not change strongly across elevations. The observed patterns in FD and PD arise due to the absence of rhinolophid bats at higher elevations. Our results are consistent with environmental filtering being a driver of community assembly, as discussed below.

A global meta-analysis of the elevational distribution of bats (that did not include data from the Himalaya) predicted that water availability and temperature drive elevational species richness patterns of bats^3^. Therefore, species richness declines linearly in mountains with wet and warm bases^3^. We uncover a trend consistent with this prediction (Fig. 3a), paralleling other tropical elevational gradients e.g.: Manu National Park in Peru^4^, Mount Kilimanjaro in Tanzania^56^, Mount Nimba in tropical West Africa^26^ and eastern Brazilian gradients^27,57^. However, it must be noted that our gradient starts from 1500 m asl instead of the foothills. Due to the complex topology of the Himalaya, it is hard to locate a single contiguous gradient from the foothills to above 3000 m. Therefore, our data do not adequately resolve whether SR declines monotonically across the full elevational range or exhibits a decline from a mid-elevation peak at 1500 m. The decline in Simpson’s Diversity Index was non-linear (Fig. 3b) with elevation, such that it did not differ significantly between Chopta and Ansuya. This finding indicates that the community at Mandal had a few dominant species, whereas at higher elevations the dominance was less pronounced.

The observed decrease in species richness with elevation is likely due to the synergistic effect of temperature on habitat and prey availability. Indeed, the elevational gradient is characterized by a strong temperature gradient with the average minimum summer temperature at Mandal (1500 m asl) being 15 °C, whereas that at Tungnath (3500 m asl) being ~ 1 °C, with frequent sleet and hailstorms. The dominant forest types and the density of vegetation also change gradually across the gradient, ultimately transforming into alpine meadows at Tungnath. This change in habitat structure potentially influences bat roost availability^58^, as well as prey density and diversity.

We observed functional underdispersion at Chopta, which is in line with our initial prediction for high-elevation communities (Fig. 4a). Functional underdispersion in high elevation communities is observed in bats in West Africa^26^ and in the Atlantic Forest of Brazil^27^, tropical bird communities^17,18^ and plants in the western Himalaya^29^. This suggests that environmental filtering may be a driving force in structuring high elevation communities of diverse taxa in mountains, at least, in the tropical and subtropical belt. By contrast, at the lowest elevation in Mandal, we observed greater than expected FRic but FDis did not deviate from the null expectation. This pattern is consistent with the idea of niche packing, wherein an increase in species richness leads to crowding of the niche space, either due to greater specialisation, or greater overlap among the niches of community members. Niche packing is a dominant mechanism in structuring bird communities of high diversity^59,60^.

The variation of PD with elevation was not significantly different from the null expectation. Recent research on tropical montane bird communities also indicates that phylogenetic structure is a poor proxy for functional diversity^18^. Studies on Himalayan birds show that much of the contemporary diversity is a result of dispersal of Southeast Asian lineages into the Himalaya since the Miocene^61,62^. During the Pliocene to Pleistocene periods, congeneric lineages organised themselves in parapatry along elevational gradients^62^. Such a colonization pattern may result in phylogenetic nestedness, and our findings may thus be reflective of past colonization processes and the resultant nestedness. Future research may focus on investigating the colonization patterns of Himalayan bats and how these affect beta diversity of FD and PD across elevations.

Previous studies that relied solely on presence data report only functional and phylogenetic dispersion measures that do not take relative abundances into account. Because we collected abundance data, we measured abundance-weighted functional dispersion, divergence and evenness (in addition to richness). Chopta has lower FDiv and FDis than expected under the null model. Its FDiv and FDis are also significantly lower than at other locations. Lower FEve implies that the abundance distribution across sonotypes is not uniform. Sonotypes such as MS, AHN and EH are more common than others at Chopta. Additionally, these common sonotypes have intermediate trait values leading to low FDiv (Fig. S3). Mandal and Ansuya have a sizeable diversity of forest-dwelling bats. The large variation in FEve at these locations is perhaps an artefact of low detection probability in forests in comparison to edge and open habitats (Fig. S2). Although FDis is most widely used to test hypotheses related to community assembly processes, data on FDiv and FEve are important in understanding alpha functional diversity which may inform local conservation measures.

Rhinolophid bats are absent at high elevations, but diverse at low elevations, with four species detected at Mandal. FD indices are calculated using distances in the standardised multidimensional trait space of the global community (i.e. across all elevations). Hence, the removal of species inevitably changes the indices for each elevational community. By removing rhinolophids from the analysis (Fig. 4b), the global trait space became smaller in its volume (richness) leading to significantly higher FRic at Chopta. The shrinking of the global trait space also altered the relative abundance distribution across all species (*cf.* FEve) and the intermediate trait values (*cf.* FDiv). Conversely, the trends in PD indices remained statistically similar to the null expectation even after excluding rhinolophids (Fig. 5b). This implies that, in terms of phylogenetic position, species are lost randomly across the elevational gradient such that phylogenetic dispersion is maintained even at high elevations. We thereby infer that the decline in FD is strongly governed by the absence of four rhinolophid bats at high elevations, which occupy peripheral regions of the trait space (Fig. S3), in addition to belonging to the same genus. This high variation in trait space is usually driven by large differences in call frequencies of sympatric rhinolophids^63^ which also exist in the four species in our dataset (Table 1, Fig. S3). The non-rhinolophid bats in the community (of the families Vespertilionidae and Miniopteridae), although more diverse in terms of species, do not exert major effects on elevational patterns owing to their similarity in functional trait space, as well as their presence across the elevational gradient.

A limitation of our FD and PD results that requires highlighting is the potential recounting of individuals across sampling sites and locations. The relative abundances of sonotypes in our calculation were based on acoustic detections and it is highly probable that the same individuals were re-recorded across sites. While this bias cannot be eliminated, we employed a conservative measure of relative abundance using the ‘Acoustic Activity Index’ framework^37^ (as described in the “Methods” section). The FD and PD calculations employ trait values and phylogenetic composition, which do not change with relative abundance when measured unweighted. The abundance-weighted group centroids may shift, but are unlikely to drastically affect the observed patterns.

Our study—a first on any mammalian taxon from the Himalaya—joins a growing body of literature quantifying community structure in montane ecosystems. Our findings are consistent with previous work on diverse taxa, with an elevational decline in species richness, and functional under dispersion in high elevation communities. These results suggest that environmental filtering likely plays a role in structuring high elevation communities. We attribute the loss of functional diversity at high elevations to the absence of four closely-related bat species with very divergent trait values belonging to the family Rhinolophidae. These changes in functional diversity across elevations are independent of phylogenetic diversity, thereby suggesting that phylogenetic diversity cannot always be used as a substitute for functional diversity. Taken together, our data provide a comprehensive understanding of the establishment of understudied tropical and montane bat communities, based on first-hand data collected in the field. This study also opens up avenues for future research on exploring (a) the role of colonization patterns in shaping community dynamics, and (b) the mechanisms of niche partitioning in communities with high functional richness. Most importantly, these data hold relevance in understanding the impacts of range-shifts of vertebrate communities, driven by climate change. Studies on the speciation of tropical montane vertebrates show that many species are adapted to live within a narrow range of temperatures^64^. Not surprisingly, tropical species are shifting their elevational ranges faster than temperate species. As the Himalaya is warming faster than the global average^32^, our results serve as an important baseline in assessing changes in bat diversity with time and in predicting potential interspecific competition in the future.

## Supplementary Information

Supplementary Information.

## Data Availability

The datasets generated during and/or analysed during the current study are available from the corresponding author on reasonable request.
