# Supplementary material for: Functional diversity of Himalayan bat communities declines at high elevation without the loss of phylogenetic diversity
Source: Sci Rep. 2021 Nov 19;11:22556. doi: 10.1038/s41598-021-01939-3 (PMC8604957; doi:10.1038/s41598-021-01939-3)
Supplement: Supplementary file 1 — Supplementary Information. [file 41598_2021_1939_MOESM1_ESM.doc]

**SUPPORTING INFORMATION**

**
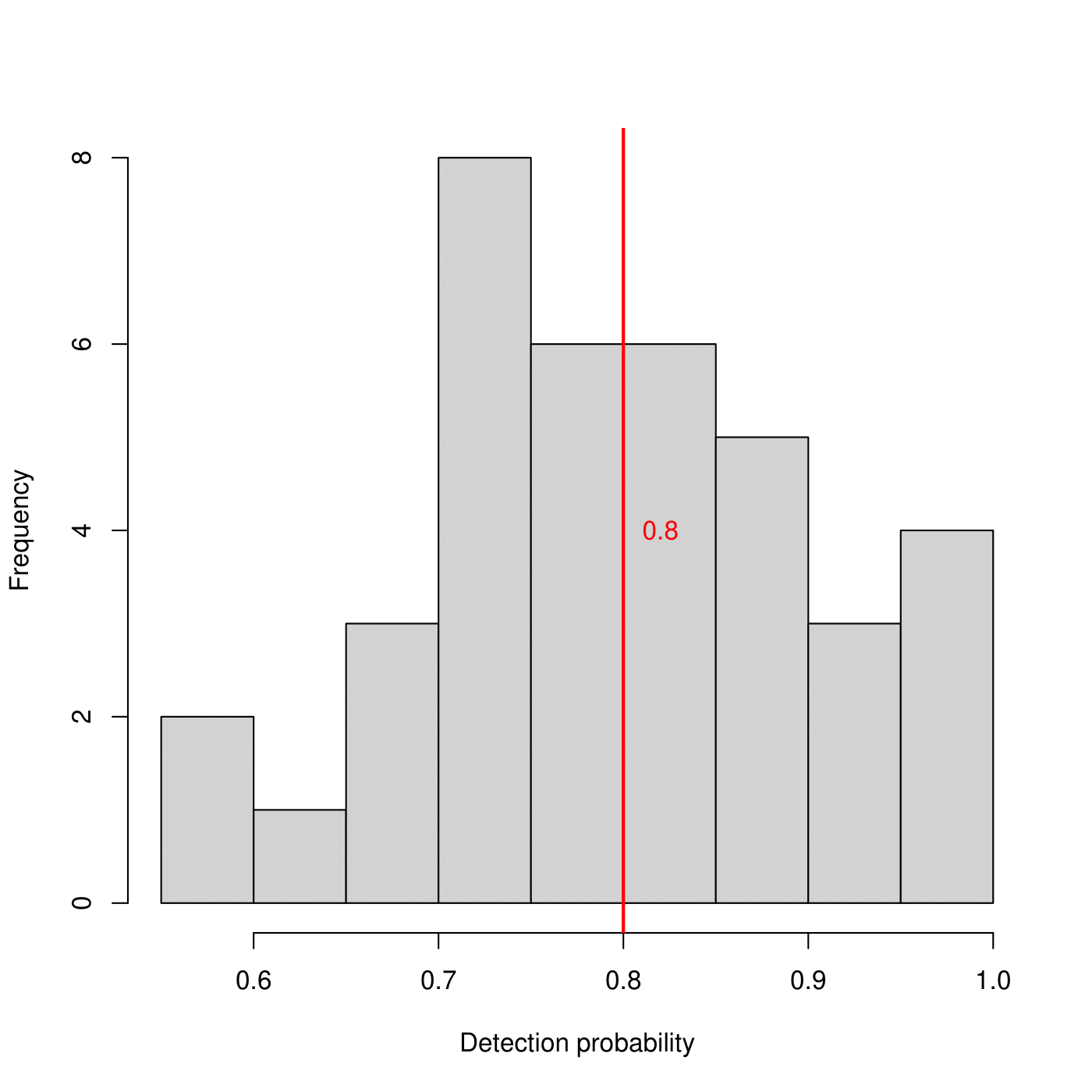
**

**Fig S1** The median detection probability calculated across all sites using mistnet captures and acoustic detections.

**
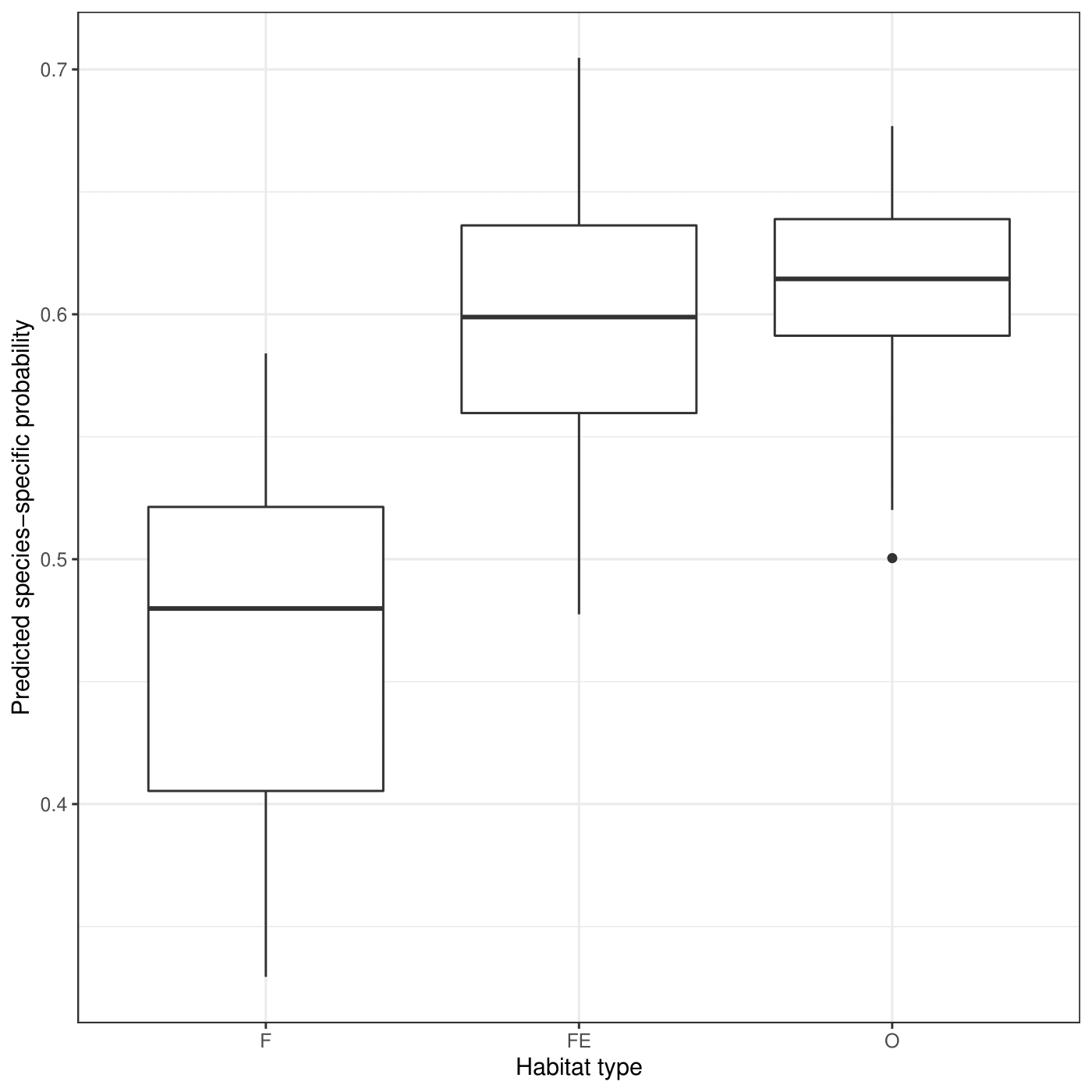
**

**Fig. S2** The heterogenity in detection probabilities across habitat types. F = forest, FE = forest edge and O = open. Species were more likely to be detected in edge and open habitats.


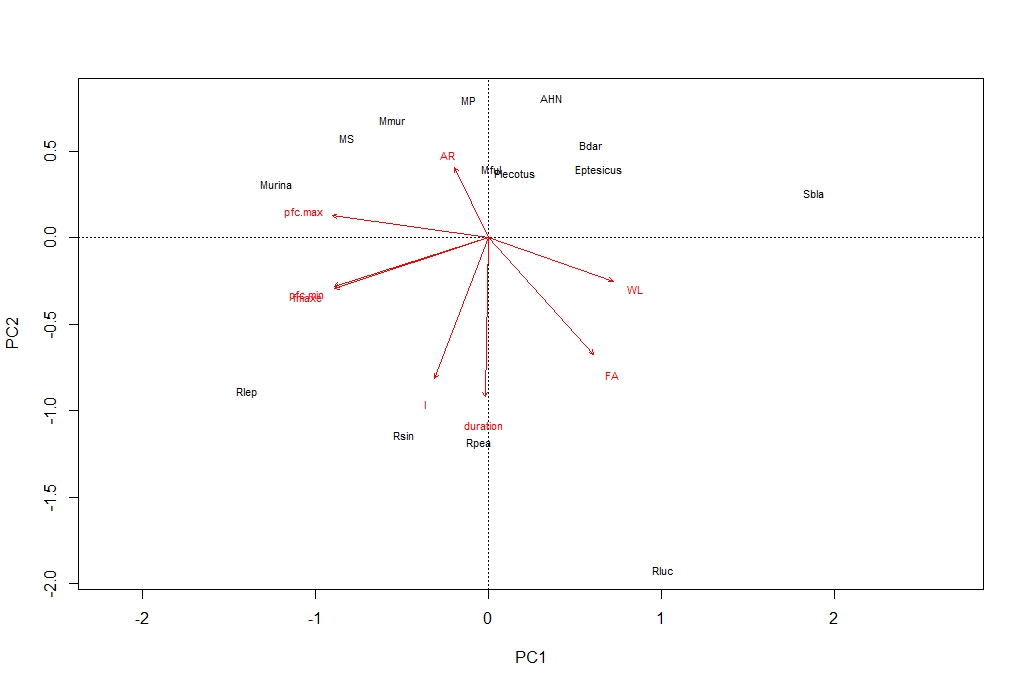


**Fig. S3** PCA ordination plot of sonotypes based on their traits listed in Table 1 of the main text. Notice the distinction between the rhinolophid bats (Rlep, Rsin, Rpea and Rluc) and the remaining bats in the assemblage.


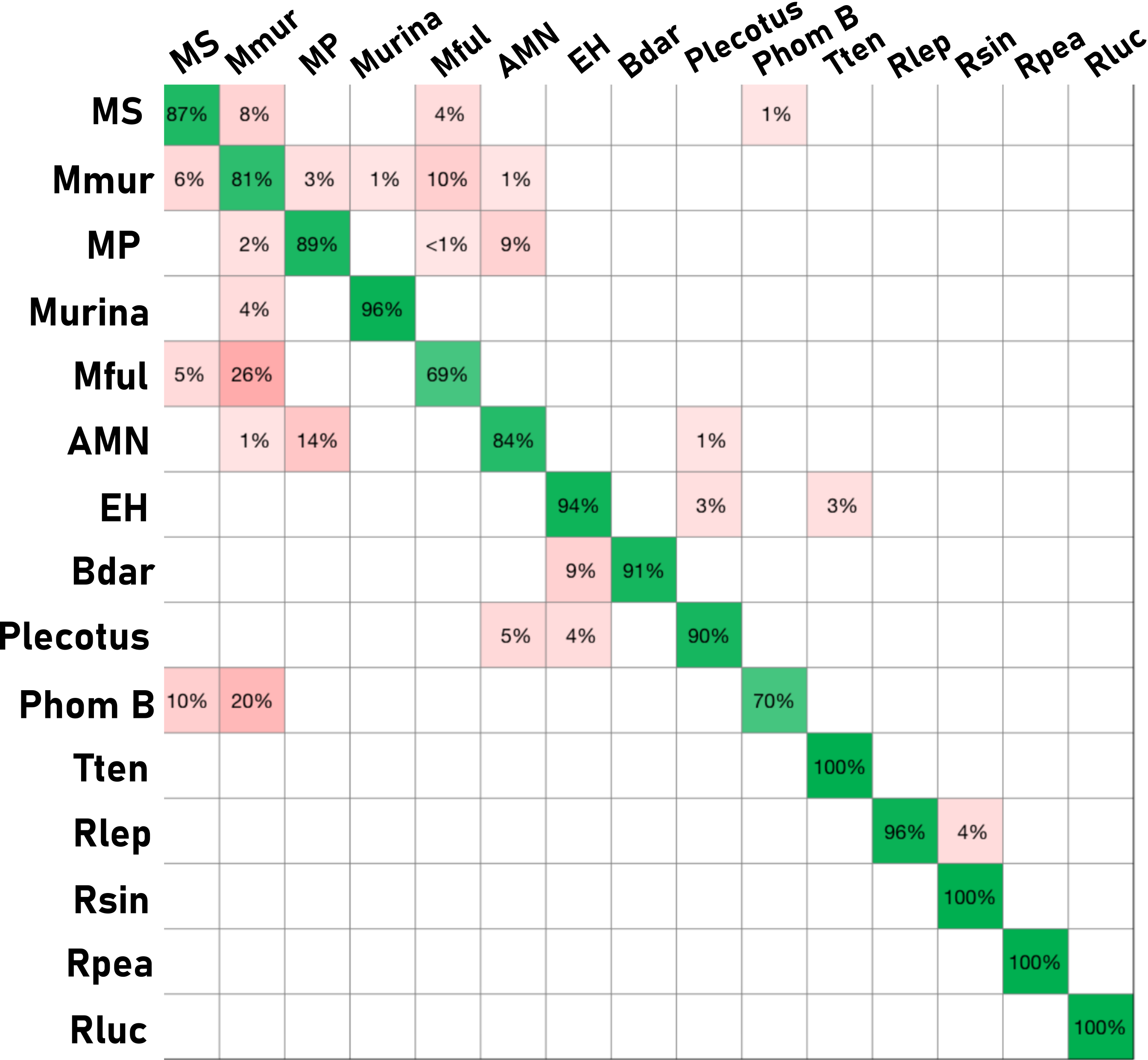


**Fig. S4** Confusion matrix showing success rates in classification of different species. Values in green show the percentage of calls that were correctly identified while the ones in red were misidentified to the corresponding species. For full forms of sonotypes, refer to Table 1. ‘*Plecotus homochrous B’* represents a high-pitched call of *Plecotus homochrous*.

**Table S1. Number of sampling points at each habitat type within each elevational location.**

| **Sampling location** | **Forest** | **Forest edge** | **Open** | **Total** |
| --- | --- | --- | --- | --- |
| **Mandal**  **(1400-1600 m)** | 3 | 2 | 3 | 8 |
| **Ansuya**  **(2000-2200 m)** | 3 | 1 | - | 4 |
| **Chopta**  **(2700-3000 m)** | 1 | 3 | 1 | 5 |
| **Tungnath**  **(3500-3700 m)** | - | - | 2 | 2 |
